# The tetraspanin CD81 mediates the growth and metastases of human osteosarcoma

**DOI:** 10.1007/s13402-019-00472-w

**Published:** 2019-09-07

**Authors:** Naoki Mizoshiri, Toshiharu Shirai, Ryu Terauchi, Shinji Tsuchida, Yuki Mori, Daichi Hayashi, Tsunao Kishida, Yuji Arai, Osam Mazda, Tohru Nakanishi, Toshikazu Kubo

**Affiliations:** 1 Department of Orthopaedics, Graduate School of Medical Science, Kyoto Prefectural University of Medicine, Kamigyo-ku, Kyoto, 602-8566 Japan; 2 Department of Immunology, Kyoto Prefectural University of Medicine, Kamigyo-ku, Kyoto, 602-8566 Japan; 3 Department of Sports and Para-Sports Medicine, Kyoto Prefectural University of Medicine, Kamigyo-ku, Kyoto, 602-8566 Japan; 4 Department of Molecular Biology and Molecular Diagnosis, Shujitsu University School of Pharmacy, Nishigawara, Okayama, 703-8516 Japan

**Keywords:** Osteosarcoma, Tetraspanin, CD81, Erk, Akt

## Abstract

**Purpose:**

CD81 is a member of the tetraspanin family of membrane proteins. Recently, it has been shown that CD81 may be involved in cancer cell proliferation and metastasis. As yet, however, there have been few reports on the expression and role of CD81 in osteosarcoma.

**Methods:**

The expression of CD81 was investigated in human osteoblast cell line hFOB1.19 and in human osteosarcoma cell lines Saos2, MG63 and 143B. The expression of CD81 was inhibited in osteosarcoma cells using siRNA after which cell proliferation, migration and invasion were assessed. We also used Western blotting to investigate the phosphorylation status of Akt, Erk, JNK and p38, and measured the expression of MMP-2, MMP-9 and MT1-MMP. In addition, we used a CRISPR/Cas9 system to stably knock out CD81 expression in 143B cells, transplanted the cells into mice, and assessed tumor formation and lung metastasis in these mice compared to those in the control group.

**Results:**

We found that CD81 was expressed in the human osteoblast cell line and in all osteosarcoma cell lines tested. The osteosarcoma cell line 143B exhibited a particularly high level of expression. In addition, we found that osteosarcoma cell proliferation, migration and invasion were decreased after CD81 inhibition, and that the phosphorylation of Akt and Erk was suppressed. Also, the expression levels of MMP-2, MMP-9 and MT1-MMP were found to be suppressed, with MMP-9 showing the greatest suppression. In vivo, we found that mice transplanted with CD81 knockout 143B cells exhibited significantly less tumor formation and lung metastasis than mice in the control group.

**Conclusion:**

Based on our findings we conclude that inhibition of CD81 suppresses intracellular signaling and reduces tumorigenesis and lung metastasis in osteosarcoma cells.

## Introduction

Osteosarcoma is the most frequent primary malignant bone tumor and occurs primarily in children and young adolescents [1]. In the 1970s, the only intervention for osteosarcoma patients was amputation, and the 5-years survival rate was < 20%. The development of chemotherapy has significantly improved the life expectancy, and the 5-years survival rate is now 65 to 70% [2]. In addition, the remarkable efficacy of chemotherapy has made the sparing of diseased limbs possible. However, despite chemotherapy, 30 to 40% of the patients experience tumor recurrence or metastasis [3]. Osteosarcoma most likely metastasizes to the lungs, and patients with comorbid lung metastasis have a poor prognosis with a 5-years life expectancy of only 20% [4]. Because current chemotherapy is not effective in at least some cases and no other effective mode of therapy has emerged in the last 40 years, new therapeutic approaches must be developed.

The tetraspanins constitute a family of membrane proteins with four transmembrane domains, and 33 types of tetraspanins including CD9, CD63, CD81, CD82 and CD151 are known in humans [5]. Tetraspanins bind with other membrane proteins to form complexes and function as molecular facilitators or molecular organizers. Moreover, tetraspanins may form tetraspanin-enriched microdomains (TEMs) on the cell membrane. Tetraspanins modulate biological processes such as cell motility, fusion and proliferation by functionally anchoring integrins and other proteins at the TEMs of various cell types for intracellular signaling [6]. Consequently, tetraspanins may also affect the invasion, proliferation and metastasis of malignant bone tumors.

CD81 is a 26 kDa member of the tetraspanin family and is expressed in various types of cells. Experiments using CD81-knockout mice have shown that CD81 is not essential for the development of T and B lymphocytes, but may play an important role in the proliferation and proper immune response of B cells [7, 8]. In addition, it has been suggested that CD81 may regulate mitosis [9] and enhance the motility and metastatic potential of hepatocarcinoma and malignant melanoma cells [10, 11]. CD81 has also attracted attention as an exosome biomarker [12] and, as such, novel anticancer drugs that can inhibit CD81 may offer therapeutic potential. It has been found that CD81 is expressed in human osteoblast (hFOB1.19) and osteosarcoma (MG63) cells [13], but a role of CD81 in osteosarcoma development has not been reported yet. Here, we report our findings on the role and molecular mechanism underlying CD81 action in osteosarcoma cells.

## Materials and methods

### Cell lines and culture

The human osteosarcoma (OS) lines Saos2 (CRL-7939), MG63 (CRL-1472) and 143B (CRL-8303), and the human fetal osteoblast cell line hFOB1.19 (CRL-11372) were obtained from the American Type Culture Collection (ATCC; Rockville, MD, USA). All cells were cultured in Dulbecco’s modified Eagle’s medium (DMEM; Nacalai Tesque, Kyoto, Japan) supplemented with 10% fetal bovine serum (FBS), 100 U/ml penicillin and 100 μg/ml streptomycin at 37 °C in an atmosphere containing 5% CO_2_. All cultures were maintained in 10 cm dishes. The OS cells were sub-cultured every 2–3 days, whereas the hFOB1.19 cells were sub-cultured every 4–5 days. After reaching 70–80% confluence, the cells were passaged using 0.05% trypsin.

### Small interfering RNA (siRNA) cell transfections

Purified siRNA duplexes targeting the human *CD81* gene (siCD81-A: 5′-CACCTTCTATGTAGGCATCTA-3′, siCD81-B: 5′-CCGCCTGTGTATATAACGTTT-3′, siCD81-C: 5′-CGCTGTGATCATGATCTTCGA-3′) were purchased from Qiagen (Valencia, CA, USA). As a negative control siNeg siRNA was obtained from Qiagen (cat. no. 1027281). To transfect the cells, the medium was discarded and replaced with antibiotic-free DMEM supplemented with 10% FBS. Next, 1.0 nM siRNA was incubated with RNAiMAX (Invitrogen, CA, USA) and Opti-MEM (Gibco, CA, USA) for 15 min, after which the cells were transfected with 1.0 nM of each siRNA in RNAiMAX, according to the manufacturer’s instructions.

### Quantitative RT-PCR

Total RNA was extracted from the cells using ISOGEN II (Nippon Gene, Tokyo, Japan) and reverse transcribed using a ReverTra Ace qPCR RT Master Mix (Toyobo, Osaka, Japan). Real-time RT-PCR was carried out using a Real-time PCR Master Mix (Toyobo) as well as probes and primers specific for the *CD81* and matrix metalloproteinase *(MMP)-2*, *MMP-9* and *MT1-MMP* genes designed by the Assays-by-Design Service (Life Technologies), using a 7300 Real-Time PCR System instrument (Applied Biosystems, Foster City, CA, USA). The amplification protocol consisted of 40 cycles of denaturation at 95 °C for 15 s, and annealing and extension at 60 °C for 1 min. The comparative threshold cycle (CT) method was used to calculate relative changes in gene expression, normalized to 18S rRNA as an internal control. Results are shown as the means of four experiments, with each sample assayed in triplicate.

### Enzyme-linked immunosorbent assay (ELISA)

Cells were seeded in 12-well plates at a density of 1.0 × 10^6^ cells/well in growth medium until they were approximately 90% confluent. Next, the cells were scraped off and diluted to a concentration of 1.0 × 10^6^ cells/ml. After centrifugation at 5000×*g* for 5 min at 4 °C, the pellets were collected and subjected to two freeze-thaw cycles to break up the cell membranes. CD81 expression was then analyzed using an ELISA kit (Cusabio Biotech Co., Ltd., Wuhan, China) according to the manufacturer’s protocol. The optical density (OD) of each well was detected using a microplate reader at 450 nm.

### Western blotting

Cells were washed twice with phosphate-buffered saline (PBS) after which proteins were extracted in RIPA buffer (Nacalai Tesque). Next, aliquots containing 35 μg protein were separated by sodium dodecyl sulfate (SDS)-polyacrylamide gel electrophoresis and transferred to polyvinylidene difluoride membranes (Hybond-P; Amersham Pharmacia Biotech, Buckinghamshire, UK). The resulting blots were incubated with the following primary human monoclonal antibodies: anti-extracellular signal-related kinase (ERK) (1:1000), anti-phospho-ERK (1:1000), anti-p38 (1:1000), anti-phospho-p38 (1:1000), anti-c-jun N-terminal kinase (JNK) (1:1000), anti-phospho-JNK (1:1000), anti-Akt (1:1000) and anti-phospho-Akt (1:1000) (Cell Signaling Technology, MA, USA). As a loading control, the blots were incubated with a mouse monoclonal anti-β-actin (1:1000) antibody (Sigma-Aldrich Co., LLC, MO, USA). After washing with TBS-Tween 20 buffer, the blots were incubated with horseradish peroxidase-conjugated secondary antibodies (1:2000) for 1 h. Chemiluminescence emission was visualized using a Chemi-Lumi One Marker (Nacalai Tesque).

### Detection of CD81 surface expression by flow cytometry

Osteosarcoma cells were seeded into 12-well plates (1.0 × 10^5^ cells/well) and incubated at 37 °C in an atmosphere containing 5% CO_2_. After 24 h, the cells were harvested and washed twice in PBS. The resulting cell suspensions (1 × 10^6^ cells/40 μl) were incubated with 25 μl CD81-PE (R&D Systems) in the dark at 4 °C for 45 min. After washing twice, 500 μl PBS was added to prepare single cell suspensions and flow cytometry analysis was performed using a FACS Vantage flow cytometer (Becton Dickinson, Franklin Lakes, NJ, USA).

### MTT proliferation assay

143B and MG63 cells were seeded in 96-well plates at a density of 5.0 × 10^3^ cells/well in 200 μl medium. Next, the cells were divided into two groups: control siRNA group and siCD81 group. Transfections with siRNA were performed the following day, as described above. Cell proliferation rates were measured every 24 h for 72 h. At each time point, 10 μl of 0.45 mg/ml 3-(4,5-dimethylthiazol-2-yl)-2,5-diphenyltetrazolium bromide (MTT; Sigma) was added to each well. After 4 h of incubation at 37 °C, 100 μl SDS was added to the MTT-treated wells to dissolve formazan crystals, and the absorption was measured at 550 nm using a spectrophotometer. Each experimental condition was analyzed in triplicate.

### Cell motility assay

Cell motility was assessed using scratch wound healing assays. To this end, 143B and MG63 cells were seeded in 24-well plates at a density of 2.5 × 10^5^ cells/well and grown until approximately 90% confluence. Next, the cells were divided into two groups: a control siRNA group and a siCD81 group. A scratch was made through each well using a CELL Scratcher (IWAKI, Tokyo, Japan). The wounded monolayers were subsequently incubated with migration assay buffer consisting of serum-free medium, after which siRNA transfection was carried out the following day. Images were captured at the same position under the microscope at 0 and 48 h. The wound healing area was calculated using ImageJ software. All samples were analyzed in triplicate.

### Invasion assay

Cell invasion assays were performed using a Cell Cytoselect 24-well cell invasion assay kit (Cell Biolabs, USA) according to the manufacturer’s protocol. Briefly, 2.5 × 10^6^ 143B or MG63 cells with 1.0 nM siCD81-A or siNeg were seeded into the upper chambers of wells in serum-free medium, whereas the lower chambers were filled with DMEM containing 10% FBS. After incubation for 48 h at 37 °C, noninvasive cells on the top surface of the membranes were removed with cotton swabs. Cells remaining on the bottom surface of the membranes were fixed with methanol for 10 min and stained with 0.5% crystal violet for 10 min at room temperature. The cells were counted in three randomly selected microscopic views. After washing, the inserts were transferred to empty wells after which 200 μl extraction solution was added. The inserts were incubated for 10 min after which the OD was measured at 550 nm.

### CRISPR/Cas9 genome editing

A CRISPR/Cas9 genome editing procedure was performed to knockout the *CD81* gene in 143B cells. To this end, a 140 bp sequence of the N-terminal *CD81* region to target double-strand breaks (Nucleic Acid Database: NM_004356) was searched. We used twenty bases of the target sequence (TGAGGTGGCCGCCGGCATCT) to knockout CD81 expression. Two complementary oligonucleotides as guide RNAs (gRNAs) were annealed and ligated into the pLentiCRISPR v2 vector (purchased from GeCKO Laboratory) containing Cas9 [14]. The resulting vectors were then transfected into HEK293FT cells (provided by Gojo Kami) for packaging, and subsequently transferred into 143B cells. The genome sequence of the edited locus in selected colonies was confirmed by sequence analysis, and real-time RT-PCR and flow cytometry were performed to assess whether CD81 was silenced in Clone KO1 and KO2 cells.

### In vivo xenograft mouse model

Four-week-old male BALB/c nude mice were purchased from Shimizu Laboratory Supplies (Kyoto, Japan). All animal experiments were approved by the Animal Care Committee of Kyoto Prefectural University of Medicine (code no. M29–137) and conducted according to institutional guidelines for animal care. 143B or 143B-KOCD81 (KO1 or KO2) cells were injected subcutaneously into the backs of nude mice (5.0 × 10^6^ cells/animal) after which tumor sizes were measured every 5 days and tumor volumes (mm^3^) were calculated using the formula: V = L × W^2^ / 2, where L and W represent the longest and shortest diameters, respectively. All mice were sacrificed 20 days after inoculation of the tumor cells, after which tumor weights were measured. For the in vivo tumor metastasis assay, 143B or 143B-KOCD81 (KO1) cells were injected into the lateral tail veins of nude mice (8.0 × 10^5^ cells/animal). After 4 weeks, whole lungs were resected. Lungs containing metastases were immersed in Bouin solution to distinguish white tumor colonies from yellowish lung parenchyma, after which the metastases were counted visually. The lungs were fixed, embedded, and sectioned for hematoxylin and eosin (HE) staining. The resulting tissue sections were imaged using Nikon software (Nikon Corporation Instruments, Tokyo, Japan) at 40× and 100× magnification.

### Statistical analysis

All experiments were performed in duplicate or triplicate. Data are expressed as means ± standard deviation (SD). Parametric one-way analysis of variance (ANOVA) was used to test for differences among groups. Tukey-Kramer tests were used to determine specific differences between groups. A *p* value < 0.05 indicated statistical significance. SPSS ver. 22 (IBM Corp., Armonk, NY, USA) was used for the analyses.

## Results

### CD81 is expressed in human osteosarcoma and osteoblast cells

**Fig. 1:**
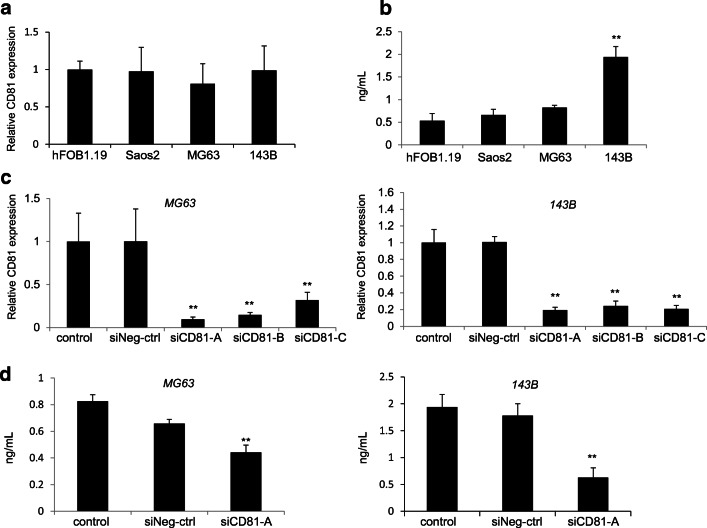
CD81 expression in cell lines and CD81 downregulation by siRNA. **a** Detection of CD81 expression in hFOB1.19 human osteoblasts and in Saos2, MG63 and 143B human osteosarcoma cells using quantitative RT-PCR. CD81 mRNA was detected in both cell types and no significant differences were observed. **b** Detection of CD81 protein expression in human osteoblasts and osteosarcoma cells using ELISA. CD81 protein was detected in both cell types. 143B cells showed a significantly higher CD81 expression than hFOB1.19 cells and the other osteosarcoma cell lines tested. **p < 0.01. **c** MG63 and 143B osteosarcoma cells were transduced with siRNA targeting CD81 (siCD81-A, -B, -C) or with control siRNA (siNeg-ctrl). Compared with control siNeg-ctrl, CD81 mRNA expression was significantly reduced by CD91-specific siRNA in MG63 and 143B cells. **d** MG63 and 143B osteosarcoma cells were transduced with siCD81-A or siNeg-ctrl. Compared with siNeg-ctrl, CD81 protein expression was significantly reduced by the CD81-specific siRNA in MG63 and 143B cells. **p < 0.01

We investigated CD81 expression by measuring CD81 mRNA and protein levels in a human osteoblast cell line (hFOB1.19) and in three human osteosarcoma cell lines (Saso2, MG63 and 143B). Using quantitative RT-PCR we observed no significant differences between CD81 expression in hFOB1.19 and in Saso2, MG63 and 143B cells (Fig. 1a). Next, CD81 protein expression was determined using ELISA, and we found that CD81 was expressed in all the cell lines tested (Fig. 1b). Particularly, we found that the level of CD81 expression in 143B cells was significantly higher than that in hFOB1.19 cells and the other osteosarcoma cell lines. In addition, we noted that the amount of CD81 expression increased with the propagation potential of the three osteosarcoma cell types.

### siRNA-mediated CD81 expression inhibition in osteosarcoma cells

We used MG63 cells, which have been reported to express CD81 and 143B cells, which exhibited the highest level of CD81 expression among the osteosarcoma cells tested, to evaluate the knockdown efficiency. We also transfected MG63 and 143B cells using three types of siCD81 molecules and a negative control, after which we measured the CD81 mRNA levels after 48 h. We found that all three siCD81 molecules (siCD81-A, siCD81-B, and siCD81-C) significantly inhibited CD81 mRNA expression in all the cell types tested. In particular, we found that the greatest inhibition efficiency was achieved with siCD81-A (Fig. 1c). Therefore, we selected siCD81-A for our further experiments. Moreover, we found by ELISA in MG63 and 143B cells that siCD81-A significantly suppressed CD81 protein expression in both cell types (Fig. 1d).

### CD81 expression downregulation suppresses the proliferation, migration and invasion of osteosarcoma cells

**Fig. 2:**
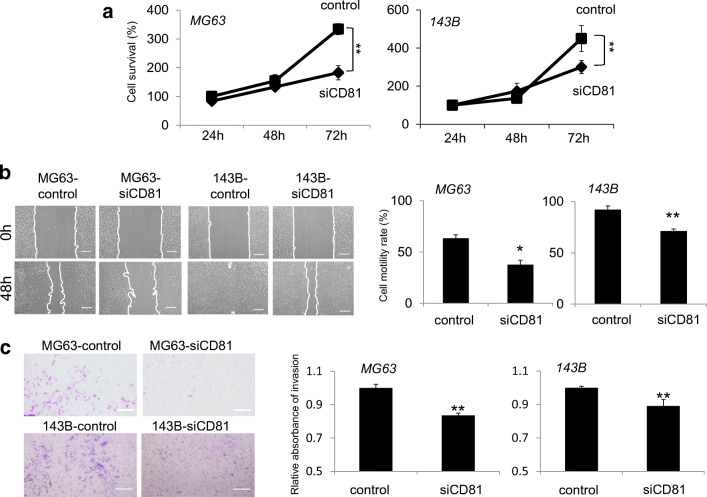
Effects of CD81 knockdown on osteosarcoma cell proliferation, migration and invasion. **a** Cell proliferation was determined by MTT assay at 0, 24, 48 and 72 h after transfection with negative control siRNA (control) or siCD81. Compared with the control, cell proliferation in MG63 and 143B cells at 72 h was significantly reduced after transfection with siCD81. **b** Scratch wound-healing assay in MG63 and 143B cells transfected with control siRNA or siCD81. Representative images of MG63 and 143B cell migration at 0 and 48 h after transfection with negative control siRNA (control) or siCD81 are shown. The migration rate was determined by measuring the area after movement. Compared with the control, cell migration in MG63 and 143B cells at 48 h was significantly reduced after transfection with siCD81. Scale bar = 500 μm. **c** Representative images of invasion assay of MG63 and 143B cells at 0 and 48 h after transfection with negative control siRNA (control) or siCD81. The invasion rate was determined by measuring absorbance. Compared with the control, cell invasion in MG63 and 143B cells at 48 h was significantly reduced after transfection with siCD81. Scale bar = 200 μm. *p < 0.05, **p < 0.01

To evaluate the effect of CD81 on the tumorigenic activities of osteosarcoma cells, we transfected MG63 and 143B cells with siCD81 and a siRNA negative control, after which we measured the proliferation, migration and invasion capacities of the cells. Using a MTT assay, no significant differences were found in both MG63 and 143B cells in the first 48 h, but at 72 h the proliferation of the cells transfected with siCD81 was found to be significantly suppressed (Fig. 2a). Using a scratch wound healing assay, we found that the migration capacities were significantly suppressed at 48 h in the siCD81-transfected MG63 and 143B cells (Fig. 2b). Furthermore, using an invasion assay, we found that the invasion capacities were significantly suppressed at 48 h in the siCD81-transfected MG63 and 143B cells (Fig. 2c). Therefore, we conclude that CD81 expression downregulation suppresses the proliferation, migration and invasion capacities of osteosarcoma cells.

### Downregulation of CD81 affects growth and motility protein signaling in osteosarcoma cells

**Fig. 3:**
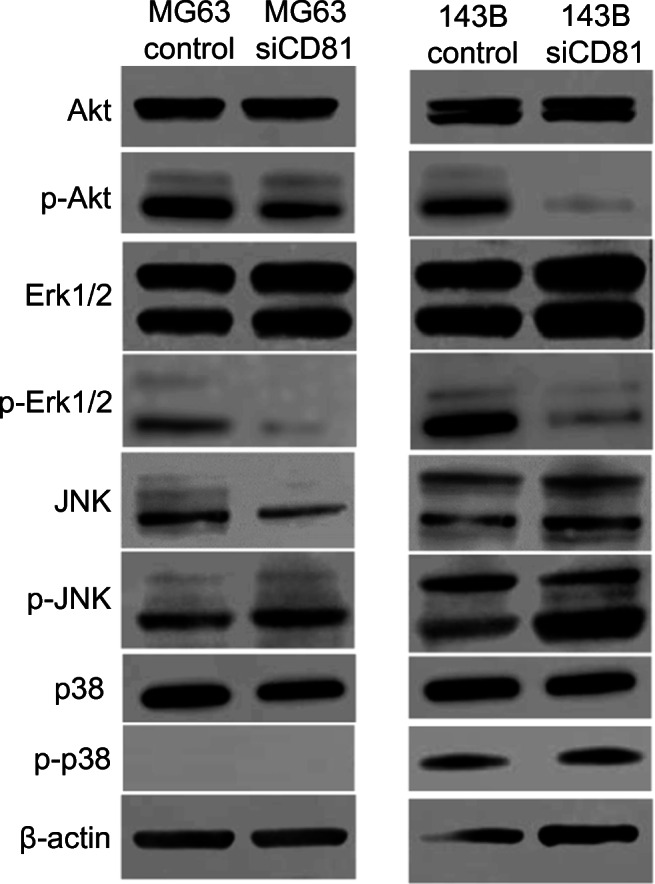
Effects of CD81 knockdown on cell signaling. Phosphorylation levels versus total protein ratios for Akt, ERK, JNK and p38 in MG63 and 143B cells were determined by Western blot analysis. Knockdown of CD81 in MG63 and 143B cells significantly inhibited phospho-Akt and phospho-ERK levels. The expression of JNK was increased, whereas that of p38 was not affected

After siCD81-mediated CD81 downregulation in MG63 and 143B cells, we used Western blotting to measure the phosphorylation of Akt, which is known to be involved in cell propagation, apoptosis and migration in osteosarcoma cells, and the phosphorylation of Erk, JNK and p38, which are proteins known to be involved in the mitogen-activated protein kinase (MAPK) pathway. We found that Akt and Erk phosphorylation in both MB63 and 143B cells was clearly suppressed. On the other hand, we found that JNK phosphorylation tended to increase slightly and that p38 was almost not phosphorylated in MG63 cells. In 143B cells we found that CD81 inhibition also had no effect on p38 phosphorylation (Fig. 3).

### Suppression of CD81 inhibits invasion factors of osteosarcoma cells

**Fig. 4:**
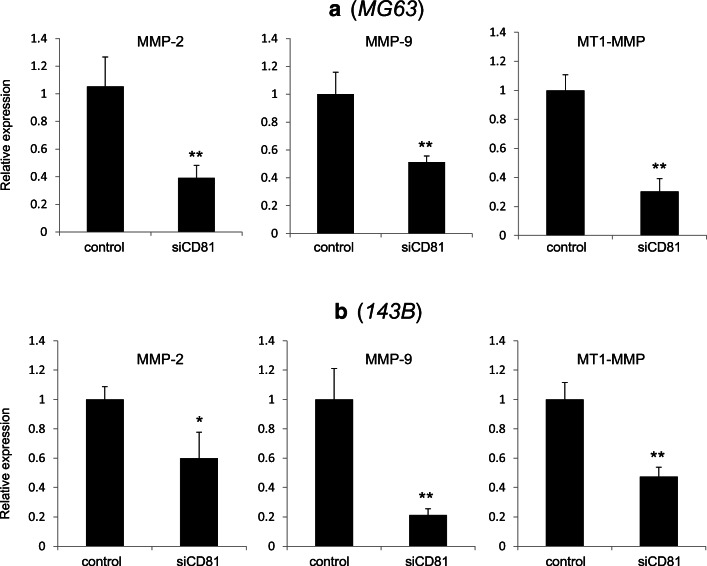
Relationship between CD81 knockdown and expression of MMP-2, MMP-9 and MT1-MMP. MMP-2, MMP-9 and MT1-MMP mRNA levels were significantly suppressed after siRNA-mediated CD81 knockdown. In both MG63 and 143B cells, the relationship between CD81 inhibition and MMP-9 and MT1-MMP expression were strong. *p < 0.05, **p < 0.01

Next, we investigated the relationship between CD81 expression and that of the matrix metalloproteinases MMP-2, MMP-9 and MTI-MMP, which are associated with invasion in osteosarcoma. We found that inhibition of CD81 in MG63 and 143B cells significantly suppressed MMP-2, MMP-9 and MTI-MMP expression (Fig. 4). In particular, we found that MMP-9 was strongly suppressed in both MG63 and 143B cells.

### CD81 knockout by CRISPR/Cas9 in osteosarcoma cells

**Fig. 5:**
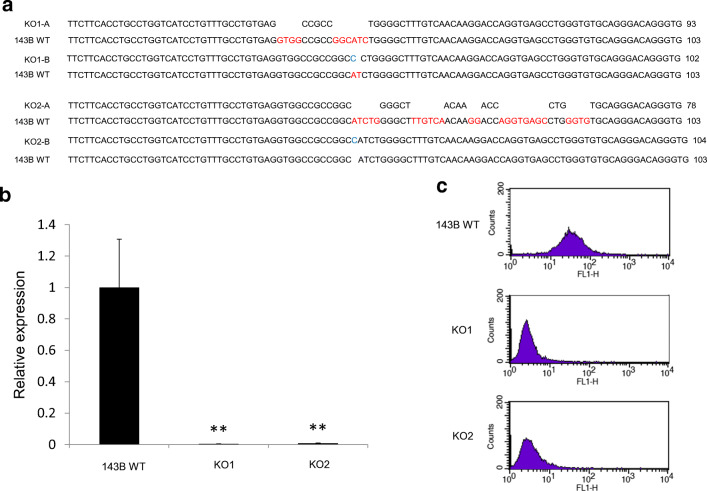
Generation of CD81 knockout osteosarcoma cells (143B) using CRISPR/Cas9. **a** The genome sequence of the edited locus was confirmed by sequence analysis. The red colored bases of 143B WT were deleted in KO1-A, -B or KO2-A, -B cells, and the blue colored bases of KO1-B or KO2-B were inserted. **b** Compared with wild-type 143B cells, CD81 mRNA expression in KO1 and KO2 cells was significantly downregulated. *p < 0.05, **p < 0.01. **c** Flow cytometry analysis showing that CD81 protein expression was significantly decreased in KO1 and KO2 cells

We permanently knocked out CD81 protein expression in osteosarcoma 143B cells using the CRISPR/Cas9 technique. After the design of a CD81 target sequence and transfection, we found that two CD81 knockout 143B cell lines (KO1, KO2) had base deletions or insertions in both alleles, designated as KO1-A and -B and KO2-A and -B (Fig. 5a). Subsequent quantitative RT-PCR revealed a significant inhibition of mRNA expression in KO1 and KO2 cells compared to 143B wild-type cells (143B WT) (Fig. 5b). Flow cytometry similarly showed a clear decrease in CD81 protein expression (Fig. 5c). These findings indicate that we successfully generated CD81 knockout osteosarcoma 143B cells using the CRISPR/Cas9 technique.

### CD81 knockout inhibits osteosarcoma propagation and lung metastasis in vivo

**Fig. 6:**
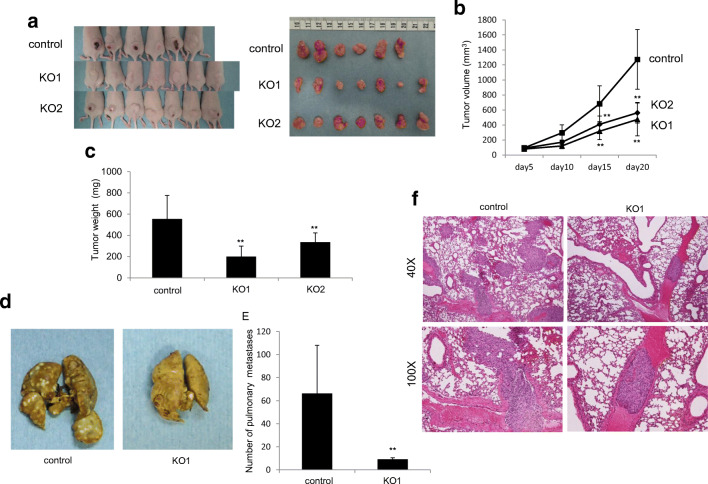
Tumorigenesis and pulmonary metastasis in nude mice inoculated with CD81 knockout osteosarcoma cells. **a** CD81 knockout 143B cells or control cells were inoculated subcutaneously into the backs of nude mice (control: *n* = 6, KO1: *n* = 7, KO2: *n* = 7). At 20 days after inoculation, mice were sacrificed. **b** Changes in tumor volumes. **c** Changes in tumor weights. **d** Representative images of pulmonary metastatic foci produced 28 days after injection of 143B control cells or CD81 knockout cells into the lateral tail vein. **e** Quantification of the number of pulmonary metastatic nodules in each group (control: *n* = 5, KO1: *n* = 5). *p < 0.05, **p < 0.01. **f** Significant micro-metastases visible in the pathological images of lung tissues of the control group (40× and 100×) on day 28 after injection

We used the CD81 knockout 143B cells generated by the CRISPR/Cas9 technique to evaluate the effect of CD81 expression on osteosarcoma propagation and lung metastasis in vivo. An in vivo propagation model was generated by transplanting mice subcutaneously with osteosarcoma cells. By doing so, we found that the CD81 knockout 143B cell groups (KO1 and KO2) exhibited significantly less tumor propagation than the 143B WT (control) group (Fig. 6a). No clear difference was found on day 5 after subcutaneous transplantation of the osteosarcoma cells, but differences gradually appeared from day 10 on, and became statistically significant starting on day 15 (Fig. 6b). Both the volume and the weight of the tumors resected on day 20 were significantly lower in both the KO1 group and KO2 group (Fig. 6b and c). These findings indicate that CD81 is involved in osteosarcoma propagation. To evaluate the potential involvement of CD81 in lung metastasis, we additionally generated a metastasis model by injecting osteosarcoma cells via the caudal vein. After 28 days the mice were sacrificed and the lungs were examined. We found that the number of lung metastases was clearly lower in the KO1 group than in the control group (Fig. 6d). Moreover, metastases were counted visually by two experienced researchers (NM and TS) and by doing so, the count in the KO1 group was found to be significantly lower (Fig. 6e). Subsequent HE staining revealed a larger number of micro-metastases in the control group than in the KO1 group. Moreover, it was noted that in the control group intravascular osteosarcoma cells were invading the lung parenchyma through the vascular walls. Contrary, in the KO1 group, the cells remained within the vessels and no invasion of the lung parenchyma through the vascular walls was observed (Fig. 6f). Together, these findings indicate that CD81 is involved in the propagation, metastasis and invasion of osteosarcoma cells in vivo.

## Discussion

It has been reported that CD81 is expressed in various types of cancer cells, forms complexes with other tetraspanins and integrins, and plays a role in the propagation, invasion and metastasis of cancer cells [9]. Here, we found that CD81 mRNA and protein are expressed in osteosarcoma cells and that the level of CD81 protein expression in these cells rises as their proliferation and invasion rates increase [15]. Therefore, we suspect that such expression may correlate with the degree of malignancy in osteosarcoma.

CD81 is known to be involved in cell proliferation mediated by the Erk/MAPK signaling pathway [10, 16, 17]. Moreover, CD81 has been found to promote integrin-mediated tumor growth in squamous cell carcinoma [18], and to be involved in the growth of both lung and breast tumors [19]. It has also been shown that CD81 is involved in the proliferation of various carcinomas, but as yet there have been no reports on osteosarcoma cell proliferation. Here, we found that the propagation of osteosarcoma cells was significantly decreased when CD81 was inhibited in these cells. Moreover we found that, as in the case of hepatocellular carcinoma, CD81 inhibition suppressed Erk phosphorylation. Therefore, we believe that CD81 may control proliferation also in osteosarcoma cells by modulating Erk phosphorylation. Furthermore, it has been found that phosphorylation and activation of JNKs may induce apoptosis and suppress proliferation in tumor cells [20, 21]. Here, we observed a slight increase in JNK phosphorylation when CD81 was inhibited. This suggests that CD81 may also play a role in osteosarcoma proliferation.

CD81 has been found to increase cell motility and invasion by elevating Akt-dependent MT1-MMP levels in melanoma [11] and by increasing integrin-mediated cell migration in ES and dendritic cells [22, 23]. In addition, it has been shown that a CD81/CD9 complex promotes integrin- and PKCα-mediated migration in breast cancer cells [24]. Therefore, CD81 is suspected of participating in cancer metastasis. Here, we found that CD81 knockdown in osteosarcoma cells suppressed their migration and invasion in vitro. Therefore, we believe that molecular mechanisms similar to those acting in other types of cancer cells may also be involved in osteosarcoma cells. Integrins, MMP-2, MMP-9, MT1-MMP and other molecules are known to be involved in the migration and invasion in osteosarcoma cells. In addition, it has been reported that MMP-2, MMP-9 and MT1-MMP may be modulated via the PI3K/Akt and Erk1/2 signaling pathways [25–28]. The extracellular matrix (ECM) serves as a barrier for the metastasis of cancer cells. MMPs are enzymes that degrade the ECM, and as such they play an important role in the invasion and metastasis of cancer cells. Twenty-three types of MMPs have been identified in humans [29] of which MMP-2 and MMP-9 degrade collagen IV, which is particularly important in the ECM [30], and MT1-MMP activates MMP-2, which is expressed on the surfaces of cancer cells [31]. In osteosarcoma, the prognosis becomes poorer as MMP-2 expression increases [32, 33]. It has also been reported that MMP-9 is expressed at high levels in osteosarcoma, and that proliferation, metastasis and migration increase as the level of MMP-9 expression increases [34, 35]. It has also been reported that MMP-2, MMP-9 and MT1-MMP are expressed at higher levels in 143B cells than in other osteosarcoma cell lines [36, 37]. Here, we found that the activation of Akt and Erk, which are known to be involved in migration and invasion, was suppressed, and that the expression of MMP-2, MMP-9 and MT1-MMP was significantly downregulated by inhibiting the expression of CD81. These findings indicate that through modulation of Akt and Erk phosphorylation, CD81 affects the expression of MMP-2, MMP-9 and MT1-MMP and, thereby, regulates osteosarcoma cell invasion. In addition, we found that CD81 may be involved in lung metastasis of osteosarcoma cells in vivo. The levels of MMP-2 and MMP-9 expression have been found to correlate with life expectancy in osteosarcoma patients [32–35]. Therefore, we believe that inhibition of CD81 expression in osteosarcoma cells may downregulate the expression of MMP-2 and MMP-9 and, thereby, improve clinical outcome.

Jinek, et al. reported in 2012 that sequence-specific cleavage of any DNA sequence is possible in vitro using a sgRNA and Cas9 [38]. Subsequently, Cong, et al. reported that the CRISPR/Cas9 system can be applied to human and murine cells [39]. With the CRISPR/Cas9 system, target DNA can be cleaved to cause a frame shift mutation and, thus, a functional knockout [14]. It has been reported that this system is also effective for the study of carcinomas and sarcomas [40], including human osteosarcomas [41, 42]. Here, we knocked out CD81 at both the mRNA and protein level by manipulating the CD81 gene sequence using a CRISPR/Cas9 system in human osteosarcoma 143B cells. We found that, by doing so, the tumor-forming capability of these cells was suppressed in vivo, and we believe that the mechanism of action is the same as in vitro. In addition, we found that metastasis to the lungs of these cells was significantly suppressed in vivo. Through pathological observations, we noted that the osteosarcoma cells in the control group invaded through the vascular walls and formed metastatic lesions in the lung parenchyma. In the CD81 knockout group we found, however, that he osteosarcoma cells did not invade through the vascular walls. Instead, they remained inside the vessels and almost no metastatic lesions in the lung parenchyma were observed. We believe that, because the MMP-mediated invasive potential was suppressed by CD81 inhibition, invasion of the lung parenchyma was suppressed and, thus, metastasis formation was decreased in the CD81 knockout group. Although we did not investigate in detail the effect of CD81 inhibition on the downstream Erk, JNK and Akt signaling pathways, it is clear that CD81 is involved in activation of Erk, JNK and Akt, and in the modulation of MMPs. Osteoblasts also express CD81 and, hence, there is a possibility that suppression of CD81 may adversely affect bone metabolism. Thus, further studies using clinical specimens are necessary. Also, interactive relationships between CD81 and integrins in osteosarcoma should be assessed in future studies.

Based on our observations, we propose that it may be possible to suppress tumor growth and metastasis, and improve prognosis in osteosarcoma patients by developing monoclonal antibodies and/or small molecular drugs that can inhibit CD81 expression.
